# Implementing a Social Determinants Screening and Referral Infrastructure During Routine Emergency Department Visits, Utah, 2017–2018

**DOI:** 10.5888/pcd17.190339

**Published:** 2020-06-18

**Authors:** Andrea S. Wallace, Brenda Luther, Jia-Wen Guo, Ching-Yu Wang, Shawna Sisler, Bob Wong

**Affiliations:** 1 University of Utah, College of Nursing, Salt Lake City, Utah

## Abstract

**Purpose and Objectives:**

Emergency departments see a disproportionate share of low-income and uninsured patients. We developed and evaluated a process for identifying social needs among emergency department patients, for facilitating access to community-based resources, and for integrating clinical and community-based data.

**Intervention Approach:**

We leveraged an academic–community partnership to develop a social needs screening tool and referral process.

**Evaluation Methods:**

In a 25-day feasibility trial incorporating rapid improvement cycles, emergency department staff screened 210 patients for social needs. Observational and interview notes were analyzed, and data were linked from patient screenings, the United Way of Salt Lake 2-1-1 consumer information system, and electronic health records.

**Results:**

Domains uncovered during pilot testing included screening based on appearance or insurance; discomfort asking stigmatizing questions; and lack of clarity regarding the screening’s purpose. During the trial, 61% (n = 129) of patients reported 1 or more need, 52% (n = 67) of whom wanted follow-up. Of the 65 patients with complete data who wanted referrals, 49% (n = 32) were ultimately reached by 2-1-1, which provided an average of 4 community referrals (eg, pharmacy programs, utility assistance). Service usage 3 months before versus 3 months after emergency department index dates demonstrated that patients with social needs experienced a significant increase in emergency department use compared with those without needs (1.07 vs 1.36, *P* = .03), while patients with no needs experienced increases in primary care visits compared with those patients with unmet needs (0.24 vs 0.56, *P* = .03).

**Implications for Public Health:**

We demonstrated the ability to systematically screen and refer for emergency department patients’ unmet social needs by using existing resources and to link screening results, service referral details, and health service data. However, our experiences demonstrate that widespread implementation efforts should thoughtfully address staff perceptions and patient communication challenges.

**Summary**

**What is already known on this topic?**

Self-management of health conditions does not occur in isolation but in the context of patients’ physical, social, and family environment.

**What is added by this report?**

Implementation efforts should emphasize universal social screening during routine emergency department visits, with careful evaluation for potential bias and stigma among staff, providers, and patients.

**What are the implications for public health practice?**

Universal screening, referral, and aggregation of clinical and social resource data are possible by using existing resources, but training and the views of those engaged in screening and referrals need to be carefully considered in efforts to implement universal social needs screening.

## Introduction

Although emergency department visits are characterized as high acuity, up to 25% of patients visiting emergency departments view them as their usual source of care (1–4) because of convenience and because of referrals from and barriers to primary care (5–7). Consequently, cost-saving efforts have been directed toward decreasing emergency department visits by increasing access to lower-cost options for treating low-acuity conditions. However, redirecting patients to lower-cost treatment options rarely results in significant cost savings (8,9). As a result, researchers have suggested that, in lieu of focusing solely on diverting low-acuity visits to less costly ambulatory care sites, health systems should focus on more fully integrating EDs into patient-centered health care delivery systems. A strategy proposed for long-term cost savings in EDs has been to direct resources toward developing health information technology linking emergency department clinicians with case managers and community-based services (8,10), with support for patient education, post–emergency department discharge care, and coordination with outside health care and social service providers (11).

As the only place in the US health care system where patients cannot be turned away for inability to pay, EDs see a disproportionate share of low-income and uninsured patients (12,13). A particularly important component of improving the quality of emergency department discharge may then rest with addressing the many patient-centered factors serving as barriers to effective self-management. Self-management of health conditions (eg, appointments, medications, dressing changes) does not occur in isolation but in the context of patients’ physical, social, and family environment. Studies have long found that adding patient-reported information (eg, need of assistance with activities of daily living, functional status) to clinical and administrative data improves the ability to predict poor health outcomes after hospital discharge (14,15). Similarly, social characteristics associated with emergency department revisits include homelessness and lack of income and insurance (11), suggesting that routine assessments of emergency department patients’ social characteristics may identify those at risk for poor outcomes after discharge and those who may benefit from targeted intervention (16).

The environments in which people live affect a wide range of health and quality-of-life outcomes, explaining as much as 75% of population health outcomes (17). Many variables fall under the broad umbrella of social determinants of health (SDOH), which are organized as the conditions and material attributes of place and patterns of social engagement (18,19). However, questions remain about population-level SDOH measurement and payment implications (20,21) and about how to assess and address SDOH during health service delivery. Although SDOH affect health and outcomes after emergency department discharge, there is no clear evidence base regarding the assessment of SDOH from which clinical interventions can be guided (22). The Institute of Medicine and the Center for Medicare and Medicaid Services have focused on identifying social needs and recommend that clinical systems screen for food and housing insecurity; financial strain; transportation, childcare, education, employment, and mental health needs; exposure to violence; and social isolation (23). Screening tools that include questions about social needs have predicted emergency department revisits and inpatient admissions after an emergency department visit (11). However, clinicians have raised concerns about how to best integrate social needs assessment into clinical care without sufficient understanding of its impact on patients and their access to resources, including ethical concerns such as compromising therapeutic relationships when identified needs are not addressed (24). Collectively, these findings suggest the importance of developing effective, sustainable methods for integrating both social needs assessment and referrals into routine emergency department service delivery.

## Purpose and Objectives

The purpose of this 2-phase, mixed-methods feasibility study was to develop and evaluate a process for systematically identifying social needs during routine health service delivery, for facilitating access to community-based supportive services, and for integrating existing clinical (ie, Epic) and community-based referral data systems. Our objectives were to 1) apply an evidence-based process-improvement model to develop a clear social needs assessment, referral, and evaluation process; 2) examine the feasibility of implementing the social needs assessment and referral process during routine care delivery in the emergency department; and 3) examine the nature, quality, and usefulness of associating data from the social needs assessment, a database of community-based service referrals, and select fields from electronic health records.

## Intervention Approach

Our study was conducted as part of an overarching effort to understand how to address patients’ social needs in the landscape of routine health service delivery in a large academic center emergency department serving a geographic area equivalent to 10% of the contiguous United States and nearly 50,000 patients annually. The emergency department care management team (comprising clinical nurses and social workers) has a strong presence in the emergency department, counseling patients both admitted and released to the community, providing care coordination and oversight of transitions of care, and evaluating clinical outcomes by using data extracted from Epic’s enterprise data warehouse. However, the care management team recognized its services have limited reach in understanding and addressing the SDOH of 120 patient discharges daily, particularly in understanding the SDOH of the approximately 90 patients discharged to community-based settings (vs inpatient admissions) daily.

### Preliminary work

In our preliminary work, we recognized that individual case managers and social workers were occasionally referring patients with known social needs to the United Way of Salt Lake City’s 2-1-1 service. The 2-1-1 service provides a free, comprehensive list of contact information for local providers who address common social needs. The program organizes social needs into 9 major categories: housing and utilities; food assistance; transportation needs; legal resources; mental health and addiction services; medical, dental, and vision insurance; employment services; education and training; and domestic violence and abuse. 2-1-1 Utah also offers population-specific services and seasonal referrals (eg, wintertime services). A full list of these services is available (https://211utah.org/index.php#geos-banner).

Although information about community-based resources may be accessed through the 2-1-1 website, the service is staffed 24 hours per day, 7 days per week by trained information specialists who use a Health Insurance Portability and Accountability Act (HIPAA)-compliant consumer encounter database (ServicePoint) to track consumer demographic characteristics, needs, and actions taken to link them with a resource information pool of over 10,000 services in Utah. In addition, information specialists have extensive experience anticipating and problem-solving consumer needs, are subject to routine quality oversight, and conduct post-call follow-up to help ensure consumers’ needs are addressed.

Next noted in the preliminary work was that social needs were not systematically and universally assessed or documented. Additionally, the clinical research team recognized that the 2-1-1 services and its encounter database had yet to be tested as a tool for understanding social needs, health service usage, and health outcomes. As such, we formed a social needs workgroup (Workgroup) composed of clinician–investigators (a nurse health services researcher, a nurse care–coordination expert, and a nurse informaticist), care management leadership (2 nurses, 1 administrator), emergency department staff (1 emergency department physician, the emergency department’s registration staff), and United Way 2-1-1 community resource referral service leadership (the executive director, the database manager, and information specialists).

### Adoption of a social needs screening tool

Through a series of meetings in 2017, the Workgroup identified and reviewed assessment tools and questions circulated by both payors and nonprofit organizations. During this process, which involved categorizing both the strengths and shortcomings of existing assessments, the Workgroup identified primary tenets to guide adoption of a social needs assessment in the emergency department.

On the basis of clinician concern about identifying social needs without clear referrals (24), the Workgroup agreed — first and foremost — that social needs assessment must focus on eliciting actionable information that could be readily addressed by either emergency department staff or 2-1-1 information specialists. The second tenet was based on a common understanding that EDs serve a diverse patient population and that health literacy is compromised among those who are in physical, emotional, or psychological distress. As such, the Workgroup focused on identifying an assessment that followed clear communication principles (eg, incorporated a uniform scale, formatted with visual space), and was written for an audience with a low literacy level. Finally, the Workgroup recognized that the fast pace of emergency department settings, in contrast to many inpatient settings, does not allow each patient to be visited by care management staff. As a result, the Workgroup focused on identifying a screening tool and process that could potentially be patient self-administered or administered with limited assistance by emergency department staff, which might allow for various models for implementation and for greater reach.

After identifying the 3 guiding tenets for social needs assessment, the Workgroup realized that existing screening tools needed to be adapted to address 1 or more of the stated objectives. With assistance and input from both English- and Spanish-speaking 2-1-1 information specialists and a volunteer network screening for SDOH in other settings in the health system, the Workgroup first selected 10 questions from existing questions recommended by HealthLeads in their Social Needs Screening Toolkit (25). They felt that the questions selected were most responsive to emergency department patient needs, gathered information that augmented rather than duplicated current emergency department clinical assessments, and followed evidence-based practice recommendations. For example, questions about exposure to violence as recommended by the US Preventive Task Force recommendations (26) were omitted in this screening because they are already asked in all clinical assessments and completed by the admitting clinicians.

One area that the Workgroup debated was the concept of immediate risk (eg, do you need help today?) versus longer-term risk (eg, have you experienced difficulties in the past year?) and how framing questions might result in identifying different patient populations. The consensus among Workgroup members was that that framing questions in terms of risk would likely result in greater sensitivity and, thus, questions were selected in terms of assessing patient experiences over the past year versus immediate need. In keeping with developing an assessment for an audience with a low literacy, the Workgroup reframed all assessment questions to begin with “In the last 12 months.” For example, the question “Do problems getting childcare make it difficult for you to work or study?” was adapted to read “In the last 12 months, have problems getting childcare or elder care made it difficult for you to work or get to appointments?”

All questions were translated into Spanish and then revised after back translation to ensure consistent communication across the 2 languages. The document layout was selected by using clear communication and low-literacy principles. For example, although it was tempting to introduce pictures on the screening tool, our team of 2-1-1 information specialists found that they did not clearly convey the intent of the questions and that doing so was at the expense of white space (which aids readability for audiences with a low literacy level). This decision was also informed by previous studies during which patients communicated a dislike for clip art (27). Finally, the entire assessment was revised to read at a fifth-grade reading level in both English and Spanish (Table 1). The final screening tool was presented to and endorsed by the health system’s patient advisory council.

**Table 1:** Social Needs Screening Questions and Outcomes (N = 210), Utah, 2017–2018

Questions	Yes, n (%)	No, n (%)	Prefer Not to Answer, n (%)
**In the last 12 months . . .**			
Have you ever not seen a doctor because you didn't have a way to get to the clinic or hospital?	45 (21.4)	162 (77.1)	3 (1.4)
Have you needed to see a doctor but could not because it costs too much?	72 (34.3)	135 (64.3)	3 (1.4)
Did you not take medications to save money?	62 (29.5)	145 (69.0)	3 (1.4)
Did you ever feel there was not enough money for food?	69 (32.9)	135 (64.3)	6 (2.9)
Did you ever feel there was not enough money for items like clothing or furniture?	75 (35.7)	130 (61.9)	5 (2.4)
Was there a time when you were not able to pay your utility bills?	68 (32.4)	136 (64.8)	6 (2.9)
Was there a time when you were not able to pay your mortgage or rent?	69 (32.9)	133 (63.3)	8 (3.8)
Have you slept outside, in a shelter, in a car, or any place not meant for sleeping?	45 (21.4)	159 (75.7)	6 (2.9)
Have you been unemployed and looking for work?	66 (31.4)	138 (65.7)	6 (2.9)
Have problems getting childcare or elder care made it difficult for you to work or get to appointments?	27 (12.9)	175 (83.3)	8 (3.8)

## Evaluations Methods

After the social needs screening tool was developed, the Workgroup applied an evidence-based organizational improvement model (20) to develop a clear process for assessing social needs during emergency department care delivery, for referring patients to the 2-1-1 community-based call center, and for linking data collected during the process.

The Workgroup conducted meetings with 2-1-1 leadership to develop an information exchange between the emergency department and 2-1-1. This process involved developing an electronic portal via Research Electronic Data Capture (REDCap) for exporting screening tool results to the 2-1-1 system, thus making a direct referral for patients who agreed to be referred to 2-1-1. REDCap is a secure, web-based data repository allowing for HIPAA-compliant data capture and storage as well as automated and electronic export of data to external sources such as 2-1-1 (28). By using this portal, patient referrals were electronically entered into the HIPAA-compliant 2-1-1 system. Only patient contact information, including patient zip codes for pre-emptive resource identification, identified needs, and a unique, complex patient identifier were shared in the 2-1-1 system. Follow-up for social needs was then done by an appointed information specialist via telephone (or text messaging) within 48 hours of emergency department assessment. Depending on the patient’s preference, they were also contacted 1 week after the initial 2-1-1 contact to establish whether goals for community-based resources had been met (eg, whether patients were able to apply for housing assistance). Finally, 2 weeks after screening, results of the 2-1-1 follow-up contact were extracted from the 2-1-1 information system and, by using the unique identifier, linked with the initial screening results and select fields extracted from Epic’s enterprise data warehouse (eg, primary care visits, emergency department visits, hospitalizations).

With automatic, electronic means to link referrals to 2-1-1, the Workgroup identified 3 primary points of patient contact during normal care delivery during which an assessment could be made: 1) by registration staff, who visit patients after they go through the triage process, are placed in a room, and are stabilized; 2) by clinical nurses when giving discharge instructions; or 3) with registration staff at the time of check-out.

**Figure 1:**
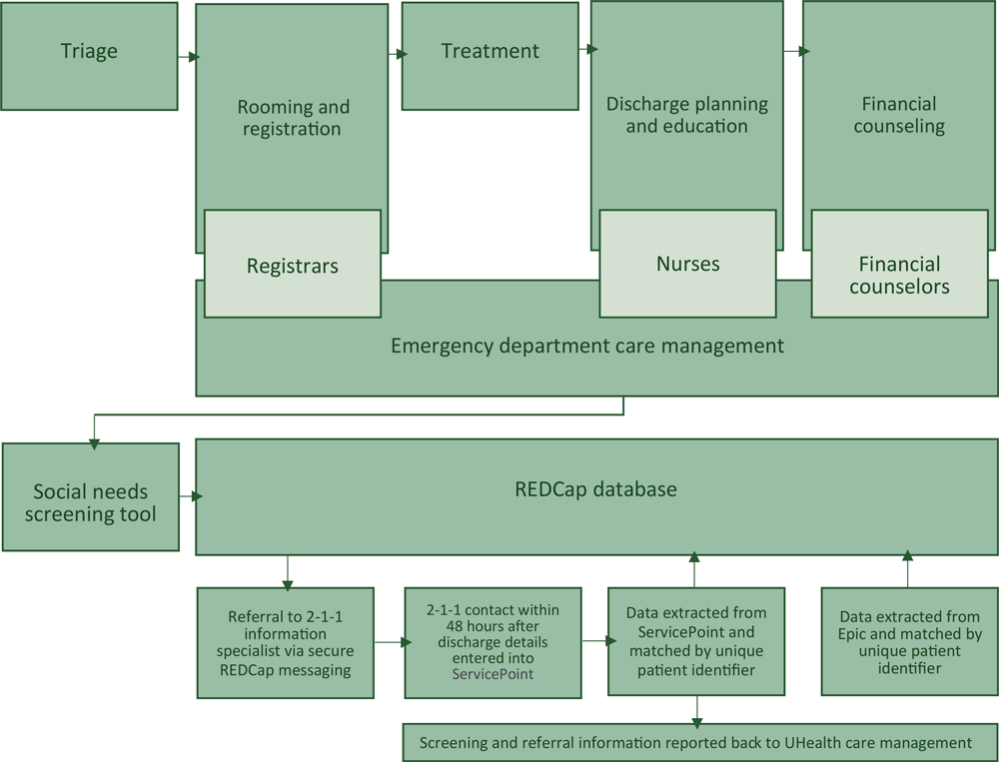
Emergency department screening and referral workflow for patients with social needs, Utah, 2017–2018. Abbreviation: REDCap, Research Electronic Data Capture database (28); UHealth, University of Utah Health.

To evaluate the 3 potential options for incorporating screening into the workflow, emergency department nursing and registration staff were observed for a total of 63 hours; 21 hours for the triage phase and 42 hours for the treatment and discharge phases. We conducted interviews during staff meetings by using an iterative process of comparing interview responses throughout the process. As a result of the collective observations, the Workgroup elected to have screening completed by the registration staff. The decision to use registration staff to deliver the screening tool was based on a strong belief that registration staff had contact with the greatest number of patients early in the admission process, which would allow screening results to be communicated to team members if immediate interventions were identified (eg, by care management, social work, or mental health specialty). The emerging workflow process resulted in a diagram demonstrating points of contact and information transfer necessary for social needs to be used as part of improving quality during emergency department service delivery (Figure 1).

Finally, the Workgroup recognized the potential for integrating screening directly into Epic. However, the team agreed that a primary emphasis of this preliminary work was to remain open to responding to early evaluation and facile in making workflow alterations. As such, the Workgroup agreed to store and merge data (ie, from 2-1-1 and the Epic data warehouse) in the REDCap data system. The Workgroup agreed that all process tests should focus on clinical implementation and scalability.

Guided by the Institute for Healthcare Improvement model (29), we conducted a series of 4-week–long pilot tests of the resulting social needs screening process, assessing for patient receptiveness; staff time and perceptions; usage of the referral system by patients discharged from the emergency department; and the quality, nature, and usefulness of data collected. In the initial 2 pilot tests, a paper screening tool was used with unique patient identifiers. In the third and fourth pilot test, the screening tool was migrated to a REDCap survey accessed by touchscreens, which allowed integration of screening into registration staff workflow because touchscreens were also newly adopted into the existing workflow for patient signatures.

## Results

Social needs screening took place during 25 days in November 2017, December 2017, May 2018, and July 2018. We collected 210 patient responses to the screening tool during these 4 phases and averaged 8.4 patient responses collected per day. Five members of the emergency department registration staff collected data. Data collected during pilot weeks 3 and 4 on touchscreens showed that screening took an average 80 seconds to complete.

### Characteristics of the study participants

Of the 210 patients completing the social needs screening questions, 129 (61.4%) indicated they had 1 or more needs. Most commonly, patients indicated not having enough money for items such as clothing or furniture (35.7%), medical care (34.3%), or food (32.9%) (Table 1). Least common were reports of childcare or elder care serving as a barrier to get to work or appointments (12.9%).

### Community-based referrals

**Figure 2:**
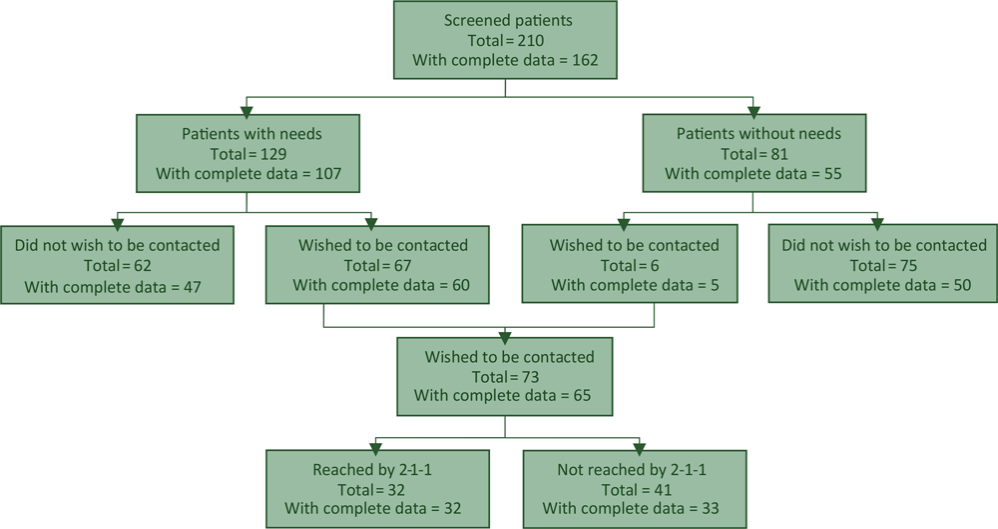
Emergency department screening and referral workflow for patients with social needs, Utah, 2017–2018.

Of the 129 patients with 1 or more stated needs, 73 (56.6%) asked for referral to 2-1-1; 32 (43.8%) were reached by 2-1-1 within 1 week of emergency department discharge (Figure 2). Patients contacted by 2-1-1 information specialists received an average of 4 service referrals. The 2-1-1 information specialists referred patients to 46 unique community resource providers via telephone and text messaging. Most commonly, patients were referred for health services (eg, community clinics, prescription drug discounts, charities covering costs of medical tests). However, referrals were also made for housing, food, utilities, children’s charities, and transportation services.

### Data associations and analysis

Creating new patient identifiers between the second and third pilot test weeks resulted in 162 patients whose data could be linked (social needs, details of 2-1-1 encounters and referrals, and health service usage data from Epic’s enterprise data warehouse). The accuracy of these data matches was confirmed by using medical records numbers, postal zip codes, and dates of birth.

Because the emphasis of this study was in feasibility and data quality versus understanding intervention effects, after the accuracy of data matching were confirmed, Wilcoxon signed rank test was used to examine number of visits (primary care provider, hospitalization, and emergency department) 3 months before versus 3 months after the emergency department index date. The analyses compared 1) patients who expressed at least 1 need (n = 107) and patients with no reported needs (n = 55); and 2) patients whom 2-1-1 attempted to contact, those who received 2-1-1 services (n = 32), and those who did not receive 2-1-1 services (n = 33). Nonparametric analyses were used because of the nonnormal distribution of count data and the small sample sizes. Means and standard deviations were reported as outcomes.

Service use 3 months before versus 3 months after the emergency department index date show that patients with at least 1 social need had a significant increase in emergency department use (1.07 before vs 1.36 after, *P* = .03) while patients with no needs had an increase in primary care visits (0.24 before vs 0.56 after, *P* = .03) (Table 2). The trend of increased emergency department visits was also noted among those who received follow-up and referrals from 2-1-1 (1.97 before vs 2.56 after, *P* = .006) (Table 3). We found no differences in hospitalizations between the 2 groups.

**Table 2:** Number of Visits 3 Months Before and 3 Months After Emergency Department Index Visit for Participants With at Least 1 Need Reported Versus No Needs Reported, Utah, 2017–2018

Type of Visit	≥1 Need, n = 107		No Needs, n = 55	
	Before, No. (SD)	After, No. (SD)	Before, No. (SD)	After, No. (SD)
Primary care provider	0.24 (0.61)	0.35 (1.09)	0.24 (0.64)^a^	0.56 (0.98)^a^
Hospitalizations	0.19 (0.62)	0.21 (0.61)	0.04 (0.19)	0.15 (0.40)
Emergency department	1.07 (3.64)^b^	1.36 (3.83)^b^	0.25 (0.70)	0.25 (0.55)

Abbreviation: SD, standard deviation.

a Significantly different at *P* = .03 level, Wilcoxon signed rank test.

b Significantly different at *P* = .03 level, Wilcoxon signed rank test.

**Table 3:** Number of Visits 3 Months Before and 3 Months After Emergency Department Index Date for Participants Who Received 2-1-1 Services Versus Those Who Did Not Receive 2-1-1 Services, Utah, 2017–2018

Type of Visit	Received 2-1-1 Service (n = 32)		No 2-1-1 Services Received (n = 33)	
	Before	After	Before	After
Primary care provider	0.22 (0.49)	0.44 (0.91)	0.21 (0.65)	0.45 (1.46)
Hospitalizations	0.13 (0.42)	0.28 (0.68)	0.15 (0.57)	0.21 (0.65)
Emergency department	1.97 (6.10)^a^	2.56 (6.27)^a^	0.58 (1.17)	0.61 (1.17)

a Significantly different at *P* = .006 level, Wilcoxon signed rank test.

### Barriers and facilitators of social needs screening and referral

Interviews with and observation of staff conducting social needs screening revealed that staff pre-emptively eliminated patients for screening based on insurance status or personal appearance or both. As appropriate, decisions to not screen were often based on diagnosis (mental health, agitation, trauma) or anticipated transfer to inpatient settings versus discharge to home. However, registration staff were observed skipping patient screenings based on the patient’s insurance status and, during interviews, said that they skipped patients who appeared well groomed or otherwise “with money.”

In follow-up interviews, emergency department team members continued to raise questions about who should be conducting social needs screenings. To many staff, it appeared that the teaching conducted by clinical nurses at the time of patient discharge addressed closely related issues such as patients’ ability to obtain medications and follow-up care. However, staff also focused on the need to incorporate screening early in the emergency department admission. Upon further questioning, staff expressed that the reason for screening early during the emergency department visit is that doing so may give case managers and other clinicians opportunity to address patient concerns that may be uncovered during the process.

Emergency department team members reported discomfort asking questions they believed to be stigmatizing. These feelings appeared to be reinforced by reports of patients who questioned the purpose of the screening questions. In response, some emergency department team members expressed desire to facilitate self-completion of patient screening questions. However, observation of the staff showed that patients were rarely allowed to self-complete, with registration staff generally asking the 10 questions of patients, largely because they viewed it as faster than waiting for patients to self-complete the screening questions.

Another observation made during data analysis was that although the screening tool was available in Spanish, all of the screenings that had complete data (ie, those with medical records data) were completed in English.

## Implications for Public Health

In this study, we demonstrated the ability to systematically screen and refer for emergency department patients’ unmet social needs by using existing resources and to link screening results, service referral details, and health service data. The resulting quantitative pilot data suggest the clinical utility of this process, demonstrating that patients who communicate 1 or more social need are those that have increasing emergency department use, which is in contrast to the increasing primary care use of those with no social needs communicated. Overall, these findings confirm that the tested processes may be a scalable solution for routine assessment of, and referral for, social needs in the emergency department. However, qualitative data collected throughout this trial also uncovered themes important to address in larger-scale implementation efforts.

Our SDOH Workgroup members echoed published concerns that universal social needs screenings should not be adopted without referral resources; linkages to community resources are critical and part of ethical screening. The solution tested in this study then went beyond initiatives focusing solely on documenting social needs during clinical care by forming new partnerships among, and linkages between, academic clinicians and existing community-based resource providers. These partnerships and linkages not only address social needs for individual patients but also lay the foundation for more rigorously examining the benefit of addressing SDOH as a follow-up to emergency department encounters. Because the United Way 2-1-1 service is a national, free-of-charge network and because HIPAA-compliant REDCap is widely available, our approach may be a sustainable and scalable alternative to proprietary services beginning to emerge in the SDOH landscape.

The pilot findings in this study document the pervasiveness of social needs in this population and that EDs may be the perfect place for addressing SDOH. The screening tool took minimal time to complete, amenable to both self-completion and verbal administration. The association between the screening results and health service usage patterns expected of vulnerable populations with social needs suggests the validity of the screening questions. However, we identified a key area of research in developing clinically relevant screening instruments that may be responsive to intervention. To date, no screening instruments are available that have been rigorously evaluated in terms of psychometrics, particularly instruments that may be feasibly completed during busy emergency department visits.

This work has several limitations. The first is that this study was conducted in 1 tertiary academic medical center with integrated case management services and in a community with a 2-1-1 with a well-supported and accessible data infrastructure. The features of this setting may not make the described processes and outcomes replicable in other less-resourced settings. A second limitation is that, while it was adapted from validated questions and questions were carefully selected based on community partner preferences, only the screening tool’s face and content validity were addressed during this study. Third, although the study team carefully reviewed observations, these may have been subject to a Hawthorne effect (ie, subjects modify their behavior because of being observed); interview themes may be biased because of existing relationships and power differentials between staff members (eg, input when managers were present during staff meetings). Finally, most of stakeholder and registration staff participants were white, and — although the screening tool was available in Spanish — all of the screenings with complete data (ie, those with medical records data) were completed in English.

Our experiences demonstrate that implementation efforts should emphasize universal screening and carefully evaluate for potential bias. Even for staff and patients who routinely discuss difficult financial questions, social needs screening in this study was a new process; thus, training needs to include discussion of communication techniques, bias, and purpose. Future research should carefully consider patient receptivity to social needs screening as well as staff comfort with questions. Although half of those with needs wished to be contacted for referrals, a substantial number of those screened refused follow-up and referrals. These questions are new to patients as part of health care encounters, especially in fast-paced clinical settings. The questions we selected are likely only the surface of what could be asked, and we do not want screening to result in unfulfilled expectations or mistrust.

We understand there are many efforts to integrate social needs screening; many support staff and volunteers are beginning to engage in efforts to address social determinants during clinical care. Experiences from this study suggest that engaging in screening as a task will not be successful without integrating it into clinical decision making and ensuring that patients view those who are engaged in referrals and follow-up as part of their health care team. The team should be credible to the patient. To accomplish this, there will have to be clarity of purpose in the screening and there will need to be technology allowing for bidirectional communication. Future efforts should focus on using technology (eg, greater incorporation of touchpads) to do the screening to assist with workflow challenges and improving data capture (eg, time to complete, information about noncompletion).
